# A Ressonância Nuclear Magnética já é um Método Adequado para Avaliação dos Resultados da Ablação de FA?

**DOI:** 10.36660/abc.20200204

**Published:** 2020-05-12

**Authors:** Cristiano F. Pisani, Mauricio Scanavacca

**Affiliations:** Hospital das Clínicas FM USP São Paulo SP Brasil Unidade Clínica de Arritmia do Instituto do Coração (InCor) do Hospital das Clínicas da FM USP (HC-FMUSP), São Paulo, SP - Brasil

**Keywords:** Fibrilação Atrial, Ablação por Cateter, Complexos Atriais Prematuros, Ressonância Nuclear Magnética/métodos, Gadolíneo

O desconhecimento da fisiopatologia da fibrilação atrial (FA) limitou por muito tempo o desenvolvimento de técnicas intervencionistas para o seu tratamento. As demonstrações de que a FA paroxística era deflagrada por extrassístoles e taquicardias oriundas principalmente do interior das veias pulmonares iniciou uma nova era no tratamento da FA. Desde então, o isolamento elétrico das veias pulmonares tornou-se o procedimento padrão na ablação da FA.^1^

A obtenção do isolamento elétrico durável das veias pulmonares tem sido o principal desafio técnico entre os especialistas, vencido paulatinamente ao longo dos últimos anos com a implementação de novas tecnologias para ablação mais efetiva, pois a principal causa das recorrências observadas nesses pacientes são as reconexões das veias previamente isoladas.^2^

O desafio tem sido maior nos pacientes com FA persistente devido a sua fisiopatologia mais complexa que envolve mecanismos adicionais pouco conhecidos, além dos focos venosos pulmonares. Admite-se que as alterações metabólicas induzidas pelo trabalho excessivo atrial durante os episódios repetitivos de FA induzam, inicialmente, o remodelamento elétrico atrial, caracterizado por alterações funcionais e transitórias dos canais iônicos das membranas celulares que modulam a atividade elétrica atrial facilitando o aparecimento de focos deflagradores em outras regiões dos átrios e condições para maior persistência em FA.^3^

A repetição e prolongamento da duração da FA evolui para o remodelamento anatômico atrial, caracterizado por alterações celulares ultraestruturais que culminam com a morte celular e sua substituição por fibrose, criando condições definitivas para o desenvolvimento de mecanismos mais complexos que mantém a FA.^4 , 5^ Paralelamente, há alteração na atividade do sistema nervoso autônomo atrial (remodelamento autonômico), outro fator facilitador para ocorrência de FA. Esse conjunto de efeitos predispõem à manutenção da FA e geram uma condição mais difícil para a recuperação do ritmo sinusal estável e permanente.^6^

Baseadas nessas informações, várias estratégias têm sido investigadas adicionalmente ao isolamento das veias pulmonares, como o isolamento da veia cava superior, da parede posterior do átrio esquerdo, do seio coronário e do apêndice atrial esquerdo, além de criação de linhas de bloqueio atrial para evitar taquicardias macrorreentrantes; tentativas de homogeneização de áreas de tecido atrial doente e a modulação do sistema nervoso autônomo atrial também tem sido implementadas.^7^ Todas essas estratégias acabam criando cicatrizes que se não forem homogêneas criarão potenciais substratos para o surgimento de novas taquicardias.^8^

Assim como na avaliação do isolamento das veias pulmonares, a principal limitação para avaliação da efetividade desses procedimentos tem sido a ausência de métodos não invasivos efetivos para avaliar a qualidade das lesões realizadas no procedimento de ablação. Até o momento, o estudo eletrofisiológico invasivo tem sido o único método capaz de demonstrar que o tecido submetido a ablação se transformou em tecido eletricamente inativo (cicatriz) eficaz no isolamento ou bloqueio da condução elétrica da área de interesse.

A ressonância magnética (RNM) do átrio esquerdo (AE) com infusão de Gadolínio e análise das áreas de fibrose pelo realce tardio tem sido considerado o método não invasivo mais promissor para avaliação da carga de cicatriz atrial dos pacientes antes da ablação, ao identificar pacientes com átrios normais e com maior probabilidade de terem procedimentos efetivos, em relação aqueles que já apresentam maior carga de fibrose e com alta probabilidade de recorrência de taquicardias atriais após o procedimento.^9^ Outro ponto interessante é que pacientes que apresentam maior extensão de fibrose atrial apresentam maior risco de eventos embólicos.^10^

Já quando a RNM é utilizada após a ablação, tem a capacidade para avaliar se as lesões térmicas promovidas pela ablação resultaram em cicatrizes definitivas e também pode identificar as falhas na formação da cicatriz ( *gaps* ), principais responsáveis pelas recorrências após ablação.^11^

Nessa edição dos ABC Correia et al.^12^ apresentam uma revisão sistemática e metanálise dos estudos que avaliaram a extensão da fibrose atrial pela RNM após a ablação por cateter de pacientes com FA. A revisão sistemática incluiu oito estudos observacionais (seis com energia de radiofrequência e dois com pacientes submetidos também à crioablação por balão). Desses, seis mostraram associação da extensão de cicatrização do AE a menor recorrência de FA após a ablação; e a metanálise que incluiu quatro estudos com 319 pacientes também confirmou que a maior extensão de fibrose atrial após a ablação, associa-se a menor taxa de recorrência de arritmias atriais (diferença média padrão = 0,52; IC 95% 0,27 – 0,76; p < 0,0001).

Esses dados são compatíveis com a expectativa de que os pacientes com maior taxa de isolamento das áreas de interesse, apresentem maior extensão de fibrose após a ablação. Entretanto, o estudo não deixa claro se o efeito benéfico foi devido a menor ocorrência de *gaps* nas lesões criadas ou se foi devido a maior extensão da ablação, por exemplo, para outras áreas como a parede posterior do AE ou septo atrial. Evidências atuais mostram que lesões extensas e controladas utilizando as novas tecnologias que produzem lesões mais efetivas e duradouras, com menos reconexões, seja com radiofrequência^13^ ou crioablação^14^ estão atualmente melhorando os resultados da ablação de FA.

Portanto, esses resultados devem ser interpretados com cautela, pois a criação de lesões atriais extensas, não homogêneas podem inclusive promover maior recorrência de arritmias atriais, especialmente as taquicardias atriais cicatriciais que em algumas situações podem até ser mais sintomáticas e de manejo mais complexo que a própria FA.^8^

Um complicador adicional é a falta de estudos demonstrando a reprodutibilidade das análises das áreas de fibrose atrial quando utilizados diferentes métodos de avaliação das imagens seja com *softwares* dedicados para processamento automático das imagens ou não. Nesse sentido, existem poucos estudos comparando as observações obtidas com a RNM com os mapas eletroanatômicos que efetivamente dirigem as ablações de FA no procedimento inicial e nas recorrências, inclusive com alguns casos sem boa concordância dos mapas com a cicatriz na RNM.^11^

Concluindo, a despeito do grande potencial que as imagens obtidas pela RNM com realce tardio com gadolínio, estudos adicionais são necessários para comprovar sua reprodutibilidade e efetividade na identificação da presença e no reconhecimento das características da fibrose atrial na seleção de pacientes que serão submetidos a ablação de FA e naqueles que já a realizaram.
