# Supplementary material for: A Ressonância Nuclear Magnética já é um Método Adequado para Avaliação dos Resultados da Ablação de FA?
Source: Arq Bras Cardiol. 2020 May 12;114(4):636–7. [Article in Portuguese] doi: 10.36660/abc.20200204 (PMC9744342; doi:10.36660/abc.20200204)
Supplement: Translation

# Translation: Is Magnetic Resonance Imaging Already an Appropriate Method for Evaluating Patients after Atrial Fibrillation Catheter Ablation?

**Authors:** Cristiano F. Pisani, Mauricio Scanavacca

**Affiliations:** Brazil Unidade Clínica de Arritmia do Instituto do Coração (InCor) do Hospital das Clínicas da FM USP (HC-FMUSP), São Paulo, SP - Brazil

**Keywords:** Atrial Fibrillation, Catheter Ablation, Atria Premature Complexes, Magnetic Resonance Spectroscopy/methods, Gadolinium

For a long time, lack of knowledge regarding the physiopathology of atrial fibrillation (AF) limited the development of interventional techniques for treating it. Demonstrations that paroxysmal AF was triggered by extrasystoles and tachycardias mainly originating from inside the pulmonary veins ushered in a new era for treatment of AF. Since then, electrical isolation of the pulmonary veins has been the standard treatment for AF.^1^ Among specialists, achieving lasting electrical isolation of the pulmonary veins has been the main technical challenge, which has gradually been overcome in recent years with the implementation of new technology for more effective ablation, given that reconnections or previously isolated veins are the main cause of recurrences observed in these patients.^2^

The challenge has been greater in patients with persistent AF, owing to its more complex physiopathology, which involves additional mechanisms that are little known, in addition to the pulmonary venous foci. It is known that metabolic alterations induced by excessive atrial work during repeated episodes of AF initially induce atrial electrical remodeling, characterized by functional and transient changes in ion channels of cell membranes that modulate atrial electrical activity, thus facilitating the appearance of trigger foci in other regions of the atria and conditions that favor increased persistence of AF.^3^

Repetition and prolonged duration of AF evolves to atrial anatomical remodeling, characterized by ultra-structural cellular changes that culminate in cellular death and substitution with fibrosis, creating definitive conditions for the development of more complex mechanisms that sustain AF.^4 , 5^ In parallel, there are changes in the activity of the atrial autonomic nervous system (autonomic remodeling), which are another factor for the occurrence of AF. Taken together, these effects predispose to the maintenance of AF, and they generate a condition where it is more difficult to recover stable permanent sinus rhythm.^6^

Based on this information, diverse strategies have been investigated in addition to the isolation of pulmonary veins, such as isolation of the superior vena cava, the posterior wall of the left atrium, the coronary sinus, and the left atrial appendage, as well as the creation of block lines in order to prevent macro-reentrant tachycardias; attempts have also been made to homogenize areas of diseased atrial tissue and to modulate the atrial autonomic nervous system.^7^ All of these strategies end up creating scars that will create potential substrates for the appearance of new tachycardias if they are not homogenous.^8^

As with the evaluation of pulmonary vein isolation, the main limitation when evaluating the effectiveness of these procedures has been the absence of effective non-invasive methods for evaluating the quality of scars induced during the ablation procedure. Thus far, invasive electrophysiological examination has been the only method capable of demonstrating that the tissue submitted to ablation has been transformed into electrically inactive tissue (scar), which is effective in isolating or blocking electrical conduction in the area of interest.

Magnetic resonance imaging (MRI) of the left atrium with gadolinium contrast and analysis of areas of fibrosis by late enhancement has been considered the most promising non-invasive method for assessing the atrial scar burden in patients before ablation, by identifying patients with normal atria and higher likelihood of having effective procedures, in relation to those who already have a higher fibrotic burden and a high likelihood of post-procedural recurrence of atrial tachycardias.^9^ Another interesting point is that patients who present greater extent of atrial fibrosis are at a higher risk of embolic events.^10^

When MRI is used after ablation, it is capable of evaluating whether thermal lesions caused by ablation resulted in definitive scarring, and it can also identify gaps in scar formation, which are primarily responsible for recurrences after ablation.^11^

In this edition of *Arquivos Brasileiros de Cardiologia* , Correia et al.^12^ present a systematic review and meta-analysis of studies that have evaluated the extent of atrial fibrosis with MRI after catheter ablation in patients with AF. The systematic review includes eight observational studies (six with radiofrequency and two with patients also submitted to balloon cryoablation). Six of these studies showed an association between extent of left atrial scarring and lower recurrence of AF after ablation, and the meta-analysis, which included four studies with 319 patients, also confirmed that greater extent of atrial fibrosis after ablation is associated with lower rate of recurrence of atrial arrhythmias (standard mean difference = 0.52; 95% CI: 0.27 – 0.76; p < 0.0001).

These data are compatible with the expectation that patients with higher rates of isolation in areas of interest will present greater extent of fibrosis after ablation. However, the study does not make it clear whether this beneficial effect was due to a lower occurrence of gaps in the induced scars or whether it was due to greater extent of ablation, for example, in other areas such as the posterior wall of the left atrium or the atrial septum. Current evidence has shown that extensive scars controlled by new technology that produces more effective and more lasting scars, with fewer reconnections, either by radiofrequency^13^ or by cryoablation,^14^ are currently improving the results of AF ablation.

For this reason, these results should be interpreted cautiously, given that the creation of extensive atrial scarring that is not homogeneous may even lead to greater recurrence of atrial arrhythmias, especially in cases of scar-related atrial tachycardias, which, in some situations, may even be more symptomatic and complex to manage than AF itself.^8^

An additional complicating factor is the lack of studies demonstrating the reproducibility of analyses of areas of atrial fibrosis when different methods of image evaluation are used, whether or not they use special software for automatic image processing. Accordingly, there are few studies comparing the observations obtained by MRI with electroanatomical maps that effectively guide AF ablations in the initial procedure and in recurrences, including some cases where there was not good agreement between the maps and the scar on MRI.^11^

In conclusion, notwithstanding the great potential shown by images obtained by late gadolinium enhancement MRI, additional studies are necessary to prove its reproducibility and effectiveness for identifying and recognizing characteristics of atrial fibrosis, both during the selection of patients who will undergo AF ablation and in patients who have already undergone the procedure.
